# Analyzing Creativity in the Light of Social Practice Theory

**DOI:** 10.3389/fpsyg.2018.02752

**Published:** 2019-01-11

**Authors:** Giuseppe Città, Manuel Gentile, Agnese Augello, Simona Ottaviano, Mario Allegra, Frank Dignum

**Affiliations:** ^1^Institute for Educational Technology, National Research Council of Italy, Rome, Italy; ^2^Institute for High Performance Computing and Networking, National Research Council of Italy, Rome, Italy; ^3^Department of Information and Computing Sciences, Utrecht University, Utrecht, Netherlands; ^4^Department of Computer Science, Czech Technical University in Prague, Prague, Czechia

**Keywords:** creativity, social practice, situational creativity, creativity of habit, TTCT

## Abstract

In this work, starting from the social practice theory, we identified two kinds of creativity: a *situational creativity* that takes place when, starting from a defined situation, a social practice is played; and a *creativity of habit* that concerns the agents' capacity for generating new practices from habit when the situation is not defined or is unexpected. To test this hypothesis, the Torrance Test of Creative Thinking (Verbal Form A) was analyzed in the light of praxeology, and the results are analyzed in a computational creativity perspective.

## 1. Introduction

Today there is growing attention toward the formalization and implementation of social skills in artificial agents. This attention arises from the importance of using agents for the accomplishment of different socially situated tasks. For example, intelligent agents, embodied in avatars, can be exploited to study social interactions (Bombari et al., 2015), and also represent a valid approach in serious games aimed at social skills training (Swartout et al., 2013; DeVault et al., 2014; Augello et al., 2016a). This demand is also evident in the area of robotics, due to the increasing use of robots as collaborators in social environments such as homes, workplaces, and schools (Breazeal, 2002; Dautenhahn, 2007). The definition of social intelligence models is essential to give such agents the ability to adapt their behavior dynamically to the different situations (Kaminka, 2013).

To this end, we must consider the theoretical perspectives that have been used in recent years to explain people's behavior in society. Apart from norm-oriented and purpose-oriented theories of actions, so-called cultural theories explain our behavior regarding structures of knowledge, which enable and drive people's interpretation of the world and require them to behave in a certain way (Reckwitz, 2002). Among these, social practice theory seems especially relevant to design a socially aware agent (Kaminka, 2013; Dignum and Dignum, 2014).

Social practices are structures of knowledge that enable a socially shared way of ascribing meaning to the world. They represent a routinized type of behavior typically performed and shared by people (for example going to work, having a meeting, and so on), that triggers our attention, affects the importance we give to our needs, and determines our expectations about the behavior of the other participants in the practice (Reckwitz, 2002).

According to Wittgenstein's reflections on the language-game notion (Wittgenstein, 1953, § 7)(Wittgenstein et al., 1969, § 519), Schatzki argues that social practices are open, temporally unfolding nexuses of actions. He points out “that fresh actions are continually perpetuating and extending practices temporally” (Schatzki, 2002), suggesting a creative dimension of actions in practices. Nevertheless, emphasizing this creative dimension of every human action does not mean to exclude the existence of rational and normative elements. On the contrary, it allows one to insert these dimensions into the practical and social contexts where an agent neither acts individually, pursuing objectives set from a means-end perspective, nor passively, internalizing and executing socially given norms (Joas, 1997).

Taking into account Hans Joas's point of view on the creativity of actions (Joas, 1997), which is different from that suggested by the so-called classical model of rationality (Searle, 2001) and the normative theories of action (Gerhardt, 2002), creativity is defined not as a dimension that is different from the action, but is a fundamental trait of it. Such a point of view suggests a perspective where social agents that perform in social contexts have a naturally creative behavior.

Social practices, therefore, must be not considered as rigid structures that strictly prescribe the agent's behavior, but, according to Wittgenstein's perspective (Wittgenstein, 1922, 1953), should be seen as a wide playing area (*Spielraum*) available to the agent. Agents who behave within social practices have the freedom to customize and extend those practices according to their personal experience and habits, or to retrieve and also combine different practices as the situation allows.

This link between creativity and social practice is also highlighted in literature on creativity. For example, Plucker et al. (2004) propose the following definition of creativity: “*Creativity is the interaction among aptitude, process, and the environment by which an individual or group produces a perceptible product that is both novel and useful as defined within a social context.”* In a similar work, Kamplis and Valtanen (2010) describe creativity as a personal skill that presumes an intentional activity that takes place in a specific context (situation), with its product possessing a value of novelty, not necessarily in absolute terms but at least for the creator him- or her-self.

To implement socially intelligent software agents a computational model was proposed (Dignum and Dignum, 2014; Dignum et al., 2015; Augello et al., 2016a). It provides a formalization that allows an agent to behave according to the appropriate social practice. However, this model does not describe how an agent can vary such a practice or combine practices. We claim that introducing some creative processes in this computational architecture can improve the agent's social behavior. Of course, it is a challenging goal, since creativity is one of the fascinating mysteries of the human mind. At the same time, the formalization and implementation of computational models is a vehicle for more extensive studies, to help us try to understand the mechanism at the base of such processes (Duch and Pilichowski, 2007; Boden et al., 2009).

We hypothesize that social practices theory is able to highlight, for each human action, the creative dimension in its complexity. Specifically, we argue that two kinds of creativity can be identified: a *situational creativity* that takes place when a social practice is performed, and a *creativity of habit* that concerns the agents' capacity for generating different practices from habit. To test this hypothesis, and with a more longer-term goal of introducing creativity in the aforementioned social agent architecture, we analyzed creativity as a primary component of social practices, by reviewing the Torrance Test of Creative Thinking (Verbal Form A) (Torrance, 1967) in the light of praxeology. The results will be used in future works to improve the social agent's framework (Augello et al., 2016a) by formalizing a computational process of creativity related to practices.

The article is structured as follows. In section 2, we explore the theoretical link between social practice and creativity presenting our research hypothesis. In section 3, we describe the method used in the study; in section 4, we interpret Torrance Test of Creative Thinking (TTCT) in the light of social practices reporting the results of our study. In section 5, we discuss the results framing them in a computational perspective based on a social practice model (Dignum and Dignum, 2014; Dignum et al., 2015; Augello et al., 2016a; Dignum, 2018)

## 2. Social Practices and Creativity: The Role of Situation and Habit

In sociology, there has long been a debate over the explanation of “social behavior.” According to a purpose-oriented theory of action (Blume and Easley, 2008), people act following individual interests which, combined, lead to social order. In contrast, in the norm-oriented theory of action (Parsons, 1968), social behavior is driven by rules that express a societal expectation. Cultural theories (Reckwitz, 2002) mediate these two different points of view, and consider an essential aspect that it is dismissed by both of them: “the implicit, tacit or unconscious layer of knowledge which enables a symbolic organization of reality” that “lay[s] down which desires are regarded as desirable and which norms are considered to be legitimate.” According to the cultural theories, the social order is achieved thanks to these collective, shared structures of knowledge which in some sense constrains us to give a meaning to the world and to act in a certain way.

In the specific case of social practices -a branch of the cultural theories- these structures of knowledge are everyday practices, routinized type of behaviors, typically and habitually performed by people, and shared and created by mutual agreements, explicit or implicit, within the society (Reckwitz, 2002).

The notion of social practices goes back to the Wittgensteinian idea that, there are no mental or physical facts that predetermine the meaning of our beliefs before our use of concepts in a social community (Esfeld, 2003) (Wittgenstein, 1953, § 154). Our behavior is conditioned by our role and the practices of interaction in the social community (Esfeld, 2003).

Practices trigger our attention to what is happening around us; they affect the importance we give to our needs, and affect our expectations regarding the behavior of the other participants in the practice (Esfeld, 2003). The rules of a practice are a rule in the Wittgensteinian sense (Wittgenstein, 1953, § 151-155, 199, 202): a generalizable procedure, the knowledge of “how to go on” in a particular context, according to our previous experiences in society. The recent definitions of social practices, formulated by Schatzki (2002) and Reckwitz (2002), takes into account this aspect to describe social practices as an organized nexus of actions performed by agents: “*Individuals, thanks to their knowledge, understanding, and expectations about situations, perform open, temporally unfolding nexuses of actions*” (Schatzki, 2002), a set of linked bodily-mental activities, established on a configuration of material, meaning, and competence elements mutually linked according to the great degrees of freedom made possible by the nature of practices.

### 2.1. Situation and Habit

**Figure 1:**
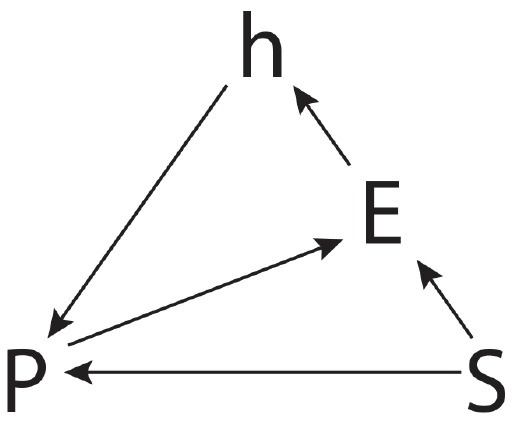
The SHaPE model.

In order to investigate the nature of practices more deeply and to characterize their intrinsic creative dimension, it is necessary to explore some concepts and their mutual relationship, in particular the key concepts of “situation” and “habit.” In literature, several descriptions are provided to clarify the subject. Bourdieu (1972), for example, describes a practice as the product of the relationship between a situation and a habit. He suggests that in the one hand a practice maintains some relationships to a situation, which has the character of immediacy and punctuality, and on the other hand, it also has a relationship with a habit, conceived as a system of lasting and transferable dispositions that, integrating all past experiences, acts at all times as a generator of perceptions, evaluations, and actions. In this regard, on our part, we have tried to summarize what is reported in the literature in an explanatory structure in Figure 1 that we call the SHaPE model (**S**ituation-**Ha**bit-**P**ractice-**E**xperience model).

The model highlights situation, habit, practice and experience as the fundamental concepts involved in theorizing creativity in the light of social practice theory. In the following lines, a more detailed description will be given. The first key concept is situation (S). According to pragmatism (Dewey, 1922), a situation represents the basic condition for carrying out each action. It is not a simple scenario of individual objects or a collection of individual objects, events, or collections of events, with which humans must deal, but is composed of elements involved in different kinds of mutual relationships. Dewey describes it as “an environing experienced world” (Dewey, 1981) where the term “environing” means that situation forms the background for a practice (P); the term “experienced” means that, in a situation, agents interact with an environment and have experience (E) of this interaction; and the term “world” proposes situation as an entity with limits and boundaries (Brown, 2012). Taking into account these descriptions, situation emerges as the essential grounding setting of a practice and of its elements. Within a situation, practices involving agents, objects, and environment can be recalled, performed and modified. Shortly, within a situation practices are played. This means that an agent, engaged in a situation, retrieves a practice and plays it (*S* → *P*), experiencing its regularities (*P* → *E*).

In this process of retrieving, playing, and experiencing a practice, the agent accords to a more-or-less conditioned freedom (Bourdieu, 1972) that allows him or her to recall not simply any practice s/he knows, but a particular practice anchored in certain constraints placed by the situation. From this perspective, the situation carries out a grounding action on a practice.

Habit (h) is the condition of the orchestration of practices (Bourdieu, 1972), the condition by which an agent plays a practice (*h* → *P*), a law deposited in each agent from the very first education. Wacquant (2016, p. 316) describes the habit as “*the ways in which the sociosymbolic structures of society become deposited inside persons in the form of lasting dispositions, or trained capacities and patterned propensities to think, feel, and act in determinate ways, which in turn guide them in their creative responses to the constraints and solicitations of their extant milieu*.” Therefore, the habit, as a system of dispositions, allows people to anticipate what is not immediately present (Määttänen, 2010), and makes them able not only to play (act and modify) individual and collective practices in a given situation but also, if necessary, to create new ones. That is, habit organizes agents' acquired practices built by past experiences, shapes current practices , conditions their perceptions of practices, and allows them to generate new practices (Bourdieu, 1979). Bourdieu suggests to conceive the habit as a corpus of semi-formalized knowledge, which determines the “reasonable” or “unreasonable” behaviour of an agent and, in defined and “ordinary” situations, enables to act or modify a practice (*S* → *P* → *E* → *h* → *P*), while, in unforeseen and undefined experienced situations, enables the agent to generate practices as possible solutions (*S* → *E* → *h* → *P*). In such a sense, some definitions given in the literature states that habit is a potential actional structure (Peirce, 1974, 6.145), a system of potentialities whose mode of being is *esse in futuro* (Peirce, 1974, 2.148), allowing agents to anticipate what is not immediately present (Määttänen, 2010).

### 2.2. Situational Creativity and Creativity of Habits

This way of thinking about the concepts of situation and habit, their mutual relationship, and the relationships they have with practices and experience, allows us therefore to explain how a social agent is able to carry out two fundamental tasks spontaneously. On the one hand, the agent is able to play, modify, and transform (more or less creatively) a practice recalled in a being-experienced situation. On the other hand, when a situation does not allow him or her to recall any practice because it is not well defined or is unexpected, s/he is able to create different possible practices on the basis of opportunities for actions, thanks to habit. These opportunities for actions can be conceived as affordances - nothing more than the set of potential environment-agent couplings (Gibson, 1979) triggered by the relationship between perception and objects (Good, 2007), which emerge as fundamental features (Chemero, 2003) and serve as evocative cues for the creation of new possible practices. This state of affairs—viewing the creative dimension as an internal feature of practices, affected by both situation and habit—led to our hypothesis about creativity in the light of the social practice theory. Specifically, we argue that if creativity is analyzed as a primary component of social practices, two possible versions of it can be identified: *situational creativity*^1^ and *creativity of habit*. The first occurs during the execution of a practice within the boundaries of a situation. For this kind of creativity, situation and the recalling of practice are at the core. The latter is observable when different practices are created thanks to the generative power of habit through opportunities for actions. Referring to this hypothesis we aim to highlight how the creative processes develop according to the concept of practice, and whether *situational creativity* and *creativity of habit* can be identified in a creative task.

## 3. Method

To reach this goal, we choose to perform a study with primary schools students. The rationale of this choice is related to the development of social skills typical of this age, which are functional to the aims of this article. Focusing on this age group allows us to analyze the creative process at a time when an agent is structuring his/her social identity (Bennett, 2011). Consequently, this choice enables the study of the specific abilities and processes of recalling, playing, modifying, and creating, more or less creatively, social practices. Moreover, this approach allows us to separate such structures from ideological superstructures (e.g., cultural, political, economic values, etc.). Even if such superstructures are present during this developmental stage, they are not at all consolidated, which makes them challenging to formalize from a computational point of view. All the statistical analysis was performed using the version 3.4.4 of the R software (R Core Team, 2018). The confirmation factor analysis and the structural equation model were analyzed using the version 0.6-1.1205 of the Lavaan package (Rosseel, 2012).

### 3.1. Participants

The study was conducted in eighteen classes of a primary school in Palermo with students in grades 3, 4, and 5. Parents of pupils were asked to read and sign an informed consent explaining the research objectives. We recruited 230 students (126 girls and 104 boys) with an average age of 9.23 years (SD = 0.92).

### 3.2. Measures

#### 3.2.1. Torrance Test of Creative Thinking

Starting from an analysis of the tools available in the literature, we selected the Torrance Test of Creative Thinking (TTCT) (Verbal Form A) (Torrance, 1967) as our principal tool of investigation. Specifically, we used the Italian version validated by Bonfiglio (1981), Tomasello (1984), and published by Sprini and Tomasello (1989). The considerable amount of research carried out through and on the TTCT in different cultures indicates that it is a tool able to identify creativity validly and reliably (Wechsler, 2006; Runco et al., 2010).

The Torrance Tests of Creativity Thinking was designed and developed by the psychologist Ellis Paul Torrance (Torrance, 1967). It measures creativity as divergent thinking ability (Guilford, 1967). Precisely, it measures a subject's ability to form heterogeneous and original reactions to certain stimuli. The test is comprised of four series of tasks: Verbal Form A, Verbal Form B, Figural Form A, and Figural Form B. The two forms, A and B, are parallel. Each one can be collectively or individually administered beginning at five years of age.

Verbal Form A of the TTCT, the series selected for our research, engages students in the following seven tasks:

1. “ASK,”
2. “GUESS CAUSES,”
3. “GUESS CONSEQUENCES”
4. “PRODUCT IMPROVEMENT”
5. “UNUSUAL USES OF CARDBOARD BOXES”
6. “UNUSUAL QUESTIONS ABOUT CARDBOARD BOXES”
7. “JUST SUPPOSE”

The “ASK” (1), “GUESS CAUSES” (2) and “GUESS CONSEQUENCES” (3) tasks stimulate students' creative skills by referring to a single image that represents a subject on all fours looking at a reflection on a surface. It looks like a child's handmade drawing, and its main feature is a lack of details so as to leave open different possible interpretations of it. The few details presented are only outlined to show their essential traits. The environmental details are reduced to the ripples of the reflecting surface, of what may be a river, and to possible wrinkles of the area on which the main subject is placed. Even the subject's physical, somatic, and clothing details are only outlined. Pointy ears and an ambiguous expression (built with the look and expression of the mouth), a pointy hat, and pointy shoes can be noted. Face, somatic features, and hat are reflected by the surface. Referring to the picture, tasks (1), (2), and (3) ask students to formulate a large number of questions, possible causes and possible consequences.

In the “PRODUCT IMPROVEMENT” task (4), students are asked to formulate a list of phrases suggesting how to improve a standard toy, a small stuffed elephant, that can be manipulated during the administration of the task.

The “UNUSUAL USES OF CARDBOARD BOXES” task (5) stimulates students to list unusual uses of cardboard boxes, and the “UNUSUAL QUESTIONS ABOUT CARDBOARD BOXES” task (6) asks them to formulate unusual questions about the boxes. Finally, in the ”JUST SUPPOSE” task (7), students have to imagine some possible consequences of an unlikely event presented in a drawing (some robes descend from clouds into a landscape where there are mountains, a valley, and a possible city).

The creativity is evaluated starting from the analysis of each sentence. The assessor has to classify the phrases according to the categories listed by the TTCT manual for each task^2^. When all the phrases have been evaluated, the following three measures can be derived for each task:

- flexibility
- fluency
- originality

Flexibility indicates the number of categories used by a student in his/her sentences ; fluency refers to the number of pertinent phrases produced; and originality is about the sum of originality values for each phrase. Such an assessment is homogeneous for the first five tasks. To support the evaluation process, the manual provides for each task a set of sample sentences with an indication of the category and the level of originality. These categories are defined on a statistical basis starting from the data collected during the validation of the test. The evaluation of tasks (6) and (7) is slightly different because the evaluation manual doesn't propose standard categories. For task (6) the flexibility cannot be calculated, while for task (7), the originality refers to the number of changes in the perspective highlighted by the assessor, sentence by sentence. Finally, the values obtained for each of the seven tasks are put together to obtain three total values of flexibility, fluency, and originality.

#### 3.2.2. Creativity Checklist

In addition to the TTCT, we selected an evaluation tool for teachers in order to have a reference measure to be used for comparison with the types of creativity proposed by this study. According to Kaufman et al. (2008), there are many available checklists to be used by teachers to evaluate students' creativity, but few have been able to produce robust validity evidence. Moreover, many creativity checklists, such as the Gifted Rating Scales (Pfeiffer and Jarosewich, 2007), are sold commercially and copyright protected. We selected the Creativity Checklist defined by Proctor and Burnett (2004) because it is a measure of creative performance within a classroom context designed for teachers and makes it possible to differentiate, during the evaluation, students' cognitive and dispositional creative traits. Both these features are effective for the aims of our work. This test, from our perspective, allows us to evaluate not only the cognitive aspects but also the non-cognitive traits that play an important role in the process of performing a social practice. Moreover, since the test is built for evaluating students in primary school, it (Proctor and Burnett, 2004, p. 425), it allows us to evaluate these traits (cognitive and not) at a time, when children are structuring their social identity (Bennett, 2011), as we mentioned above about TTCT.

The Creativity Checklist is usually administered to collect teachers' observational data about nine cognitive features and dispositional traits displayed by students engaged in classroom activities that require creativity.

The test is composed of nine items (or descriptors) of cognitive and dispositional traits, and performance indicators that discursively describe them. Specifically, the nine items assess whether each student is “a fluent thinker,” “an original thinker,” “an intrinsically motivated student,” “an elaborative thinker,” “an intrinsically motivated student,” “a curious student who becomes immersed in the task,” “a risk-taker,” “an imaginative or intuitive thinker,” and/or “a student who engages in complex tasks and enjoys a challenge” (Proctor and Burnett, 2004, p. 426).

Teachers evaluate each item according to a 3-point Likert scale (1 = rarely, 2 = sometimes, 3 = often) that rates how often the students show some creativity-linked cognitive and dispositional traits: less than 30% of the time (1 = rarely), between 30 and 70% (2 = sometimes), or more than 70% of the time (3 = often).

### 3.3. Materials

The procedure of analysis of the Torrance test requires the evaluator to analyze each sentence produced by the students, selecting the category to which it belongs and the level of originality. This process is generally guided by the sample sentences provided by the evaluation manual.

**Figure 2:**
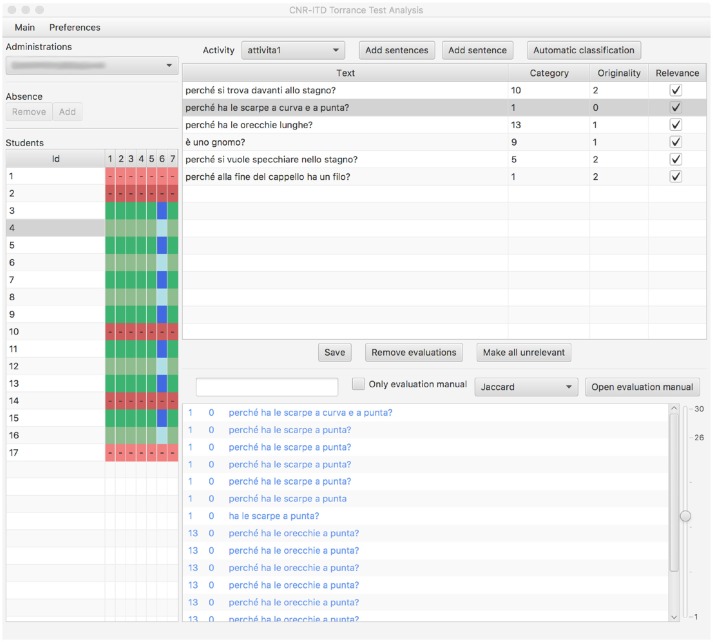
The evaluation support software for TTCT.

To support the evaluators and speed up the evaluation process, we designed a software (see Figure 2) that, for each student's production, interactively suggested a list of sentences that are semantically close, and indicated the relative evaluations regarding category and originality. The software was designed to be conformed to the indications provided by the evaluation manual.

This list is built starting from the sample sentences listed in the evaluation manual, with the addition of sentences previously analyzed by the other evaluators. The software suitably indicated the source of suggested phrases (the evaluation manual, the sets of previous evaluations) through the use of different colors.

The evaluator can obtain such lists using different methods of similarity implemented exploiting the LingPipe library (Alias-i, 2008) and in particular the *Jaccard* distance, the *Jaro-Winkler* distance, beyond the classic *TfIdf* distance. Moreover, the evaluator can perform a search by entering words similar to those listed in the sentence, or can open the list of all the examples in the manual directly.

The tool has been developed as a support for the evaluation, not as a replacement for the human operator who, starting from the suggestions of similarity proposed by the software, has the final responsibility for the insertion of the evaluation.

It is worth to highlight that the use of this software has allowed the evaluators to analyze in real time, where present, any divergence in the evaluations, allowing them to highlight and resolve these divergences collaboratively by changing, where necessary, even the evaluations provided previously.

### 3.4. Procedure

The trial was conducted according to the instructions given in the Torrance evaluation manual. The test was administered in paper-and-pencil modality, to groups that correspond to the classes involved in the trial. For each group, after a general introduction aimed at creating the right class climate, each task of the Torrance was preceded by an explanation as described in the evaluation manual. The observers remained in the classroom during the activity to mark the progress between the tasks according to the schedule, verify the correct development of the test, and support the children who requested further explanations. Each administration had an expected duration of 45 minutes, net of the time for explanations. On average, one hour was enough to complete the test. Administration in the eighteen classes took a total of about six weeks.

Subsequently, in separate sessions, the Creativity Checklist was distributed to the teachers of the classes involved. For each class, the two principal teachers (usually Primary Language and Math teachers) evaluated their children collaboratively, according to the creativity checklist scale.

## 4. Results

### 4.1. Preliminary Analysis

From the initial sample of 230 students, 222 students have completed at least one activity of the Torrance Test; of these 222, one student was not evaluated with the Creativity Checklist by the teachers because he/she moved to another school. In total, we conducted our analysis on a sample of 221 students.

Descriptive statistics (means, standard deviations, and ranges) were computed to summarize the characteristics of the tools used. Table 1 shows the descriptive measures of flexibility, fluency, and originality for each of the seven activities of the TTCT. Task (5) on average had higher values for all the three measures of creativity as well as for the standard deviation. The task (7) had lower values of fluency and flexibility, while task (1) had the lower value of originality. The values in the table show the non-normal nature of the Torrance variables. In Table 2 the Pearson correlations of Torrance Test variables are reported.

**Table 1:** Descriptive data of Torrance Test variables.

	**Min**	**Max**	**Median**	**Mean**	**Skew**	**Kurtosis**
fle1	1.00	12.00	5.00	5.32	0.30	0.39
fle2	1.00	8.00	3.00	3.32	0.29	-0.26
fle3	1.00	9.00	3.00	3.66	0.57	0.11
fle4	1.00	13.00	5.00	5.57	0.35	-0.30
fle5	1.00	17.00	7.00	7.40	0.25	-0.13
fle7	0.00	26.00	3.00	4.07	2.99	11.67
flu1	1.00	27.00	9.00	9.67	0.80	0.80
flu2	1.00	27.00	6.00	6.60	1.32	4.28
flu3	1.00	22.00	6.00	7.22	1.14	1.41
flu4	1.00	49.00	11.00	13.61	1.22	1.27
flu5	1.00	55.00	14.00	16.61	1.17	0.96
flu6	1.00	36.00	9.00	9.39	1.34	2.56
flu7	1.00	31.00	5.00	6.46	2.61	8.44
ori1	0.00	28.00	7.00	7.95	1.11	1.10
ori2	0.00	48.00	10.00	10.59	1.61	5.74
ori3	0.00	37.00	10.00	11.17	1.16	1.87
ori4	0.00	56.00	12.00	15.27	1.13	0.82
ori5	1.00	84.00	21.00	25.53	1.25	1.31
ori6	0.00	24.00	2.00	3.15	1.91	4.89
ori7	0.00	51.00	8.00	9.46	2.30	10.71

**Table 2:** Pearson correlations of Torrance Test variables.

	**fle1**	**flu1**	**ori1**	**fle2**	**flu2**	**ori2**	**fle3**	**flu3**	**ori3**	**fle4**	**flu4**	**ori4**	**fle5**	**flu5**	**ori5**	**fle7**	**flu7**
fle1																	
flu1	0.78^***^																
ori1	0.49^***^	0.73^***^															
fle2	0.27^***^	0.28^***^	0.32^***^														
flu2	0.38^***^	0.52^***^	0.44^***^	0.58^***^													
ori2	0.37^***^	0.49^***^	0.45^***^	0.57^***^	0.93^***^												
fle3	0.32^***^	0.31^***^	0.19^**^	0.33^***^	0.38^***^	0.36^***^											
flu3	0.40^***^	0.53^***^	0.31^***^	0.32^***^	0.68^***^	0.60^***^	0.69^***^										
ori3	0.40^***^	0.53^***^	0.37^***^	0.29^***^	0.64^***^	0.60^***^	0.66^***^	0.91^***^									
fle4	0.38^***^	0.33^***^	0.22^**^	0.25^***^	0.36^***^	0.36^***^	0.27^***^	0.35^***^	0.36^***^								
flu4	0.39^***^	0.37^***^	0.26^***^	0.22^**^	0.41^***^	0.42^***^	0.21^**^	0.35^***^	0.38^***^	0.73^***^							
ori4	0.31^***^	0.30^***^	0.27^***^	0.24^***^	0.40^***^	0.43^***^	0.21^**^	0.32^***^	0.36^***^	0.66^***^	0.90^***^						
fle5	0.25^***^	0.31^***^	0.20^**^	0.12	0.19^**^	0.20^**^	0.27^***^	0.31^***^	0.30^***^	0.35^***^	0.41^***^	0.40^***^					
flu5	0.35^***^	0.41^***^	0.29^***^	0.13	0.29^***^	0.28^***^	0.26^***^	0.37^***^	0.40^***^	0.36^***^	0.54^***^	0.47^***^	0.82^***^				
ori5	0.32^***^	0.39^***^	0.32^***^	0.13	0.26^***^	0.26^***^	0.27^***^	0.35^***^	0.39^***^	0.33^***^	0.49^***^	0.47^***^	0.78^***^	0.96^***^			
fle7	0.35^***^	0.32^***^	0.15^*^	0.21^**^	0.33^***^	0.29^***^	0.19^**^	0.28^***^	0.27^***^	0.41^***^	0.57^***^	0.35^***^	0.27^***^	0.42^***^	0.32^***^		
flu7	0.39^***^	0.38^***^	0.21^**^	0.23^**^	0.48^***^	0.46^***^	0.21^**^	0.40^***^	0.38^***^	0.43^***^	0.64^***^	0.48^***^	0.28^***^	0.46^***^	0.40^***^	0.80^***^	
ori7	0.38^***^	0.44^***^	0.32^***^	0.29^***^	0.41^***^	0.39^***^	0.31^***^	0.41^***^	0.41^***^	0.34^***^	0.46^***^	0.39^***^	0.27^***^	0.40^***^	0.39^***^	0.45^***^	0.78^***^

*** *p < 0.001*,

** *p < 0.01*,

* *p < 0.05*.

Regarding the Creativity Checklist, the analysis of the results exhibited a high level of classical reliability (α = 0.949). In Table 3 the Spearman correlations of Creative Checklist items are reported. Moreover, we performed a confirmatory factor analysis in order to confirm the one-dimensional structure of this instrument. The confirmatory factor analysis of the Creativity Checklist shows a good fit to data (ratio of χ^2^ to *df* = 1.9, CFI = 0.998, TLI = 0.997, RMSEA = 0.084).

**Table 3:** Spearman correlations of creative checklist items.

	**a1**	**a2**	**a3**	**a4**	**a5**	**a6**	**a7**	**a8**
a1								
a2	0.83							
a3	0.80	0.74						
a4	0.72	0.69	0.70					
a5	0.64	0.62	0.58	0.63				
a6	0.68	0.64	0.67	0.67	0.73			
a7	0.65	0.69	0.64	0.59	0.60	0.66		
a8	0.77	0.69	0.70	0.69	0.60	0.66	0.63	
a9	0.63	0.67	0.58	0.63	0.70	0.62	0.78	0.63

*All correlations are significant at the 0.0001 level*.

### 4.2. Conceptual Model Comparison

The initial hypothesis of this work is that it is possible to analyze the TTCT in the light of the theory of social practices. Analysis of the tasks that comprised the TTCT in this theoretical context allows us to link them to the hypothesized types of creativity, “situational creativity” and “creativity of habit,” by mean of the SHaPE model.

Within tasks (1), (2) and (3) a situation is given through a picture. This picture recalls the elements of a story, and in the three tasks the student is asked to produce questions for understanding what is happening, formulate hypotheses about the causes that led to that situation, and describe possible consequences, respectively. Task (7) asked students to imagine some possible consequences of a specific (albeit unlikely) event, presented in a drawing, which proposes, accordingly to tasks (1), (2), and (3), a given starting situation. From a social practices perspective, it means that, through an image, the TTCT shows some elements of the practice that students can manage more or less creatively.

Within the given situation, there are some possible actions that student can figure out and arrange as directly carried out by the character. Other possible actions can be carried out by characters that are external to the picture. When students manage situational elements (e.g., the possible consequences and causes related to the situation), they show how they manipulate their knowledge related to practice. Tasks (1), (2), (3) and (7) of the TTCT can reveal how students “create” or creatively play practices inside the situation, according to what we call *situational creativity*.

Tasks (4) and (5), instead, clearly highlight what we called *creativity of habit*. They provide students with an undefined or unexpected situation. The “PRODUCT IMPROVEMENT” task (4), puts the students in engagement with some features of the toy (its shape, color, somatic traits, etc.) that act as opportunities for action and stimulate the elaboration of possible solutions. In the language of praxeology, when students elaborate some possible improvements of the toy they generate a series of new possible practices thanks to their habit.

The same process occurs in “UNUSUAL USES OF CARDBOARD BOXES” task (5). Within a context that does not offer some fixed points, the student is stimulated to “invent” unusual uses of boxes relying only on their past experiences and dispositions. According to the social practices theory, students create new and different practices relying on the generative power of their habit In our analysis, we did not consider the task (6). It is the only task of the TTCT that is not explicitly related to the situation concept. This particularity is confirmed in the literature so that this task is often not considered in the dimensional analysis of TTCT (Krumm et al., 2016).

To verify if the re-arrangement of the TTCT in the logic of social practices theory highlights the existence of the two proposed types of creativity, we have compared four different models. The first two reproduce models present in the literature, and the remaining ones that, using the first two as their bases, provide a review according to the analysis of the tasks previously reported.

**Figure 3:**
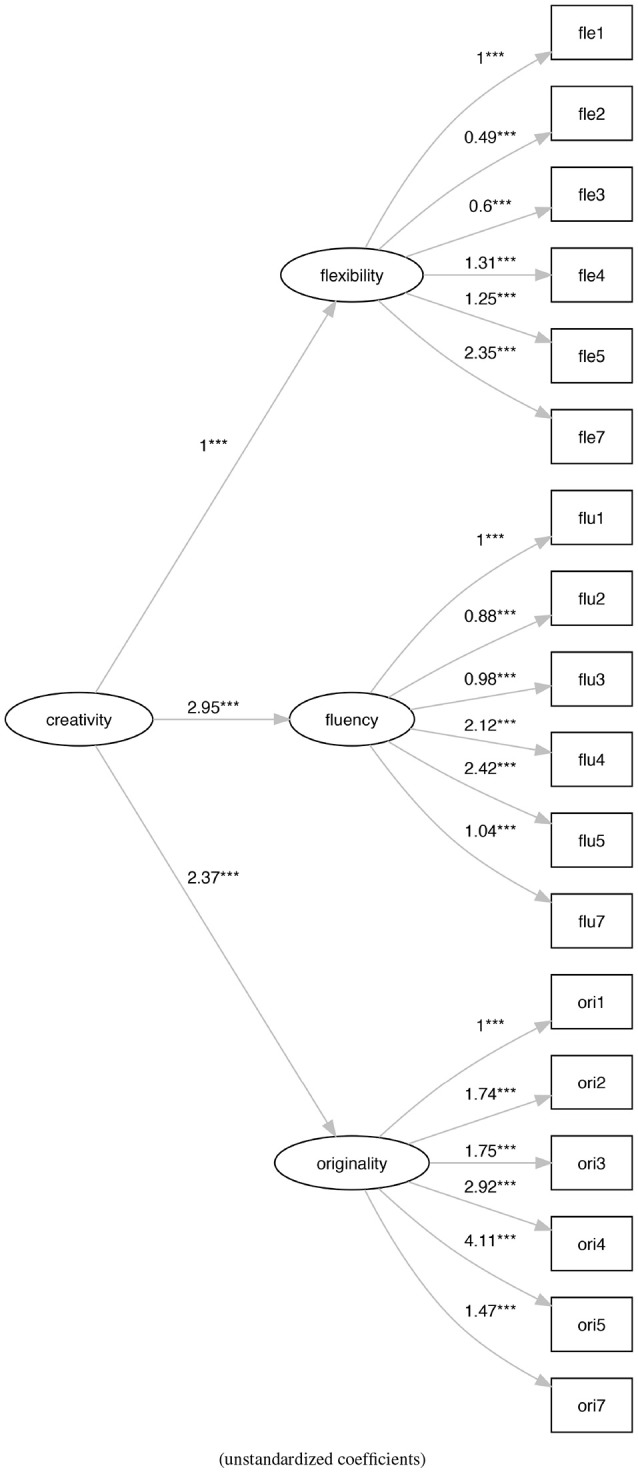
The original Torrance factor model (M0). ***p < 0.001.

The first model (M0) (Figure 3) is the original model proposed by Torrance with the three latent variables of flexibility, fluidity, and originality that contribute to the definition of a single latent variable that represents the creativity.

**Figure 4:**
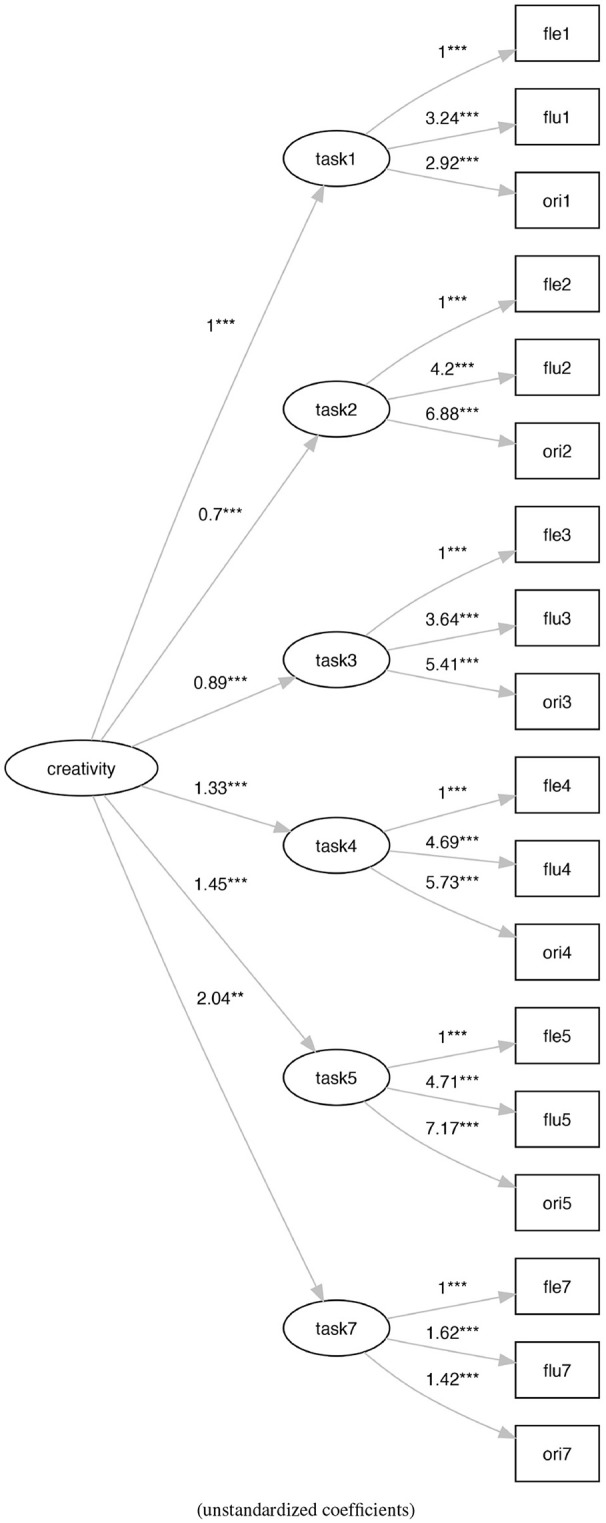
The activities-based Torrance factor model M1. **p < 0.01, ***p < 0.001.

Model M1 (Figure 4) is a re-proposition of a task-based re-arrangement of the Torrance model (Krumm et al., 2016; Humble et al., 2018) with the six task-based latent variables that contribute to the definition of a unidimensional latent variable of creativity. This model generally presents a better adaptation to the data with respect to model M0.

**Figure 5:**
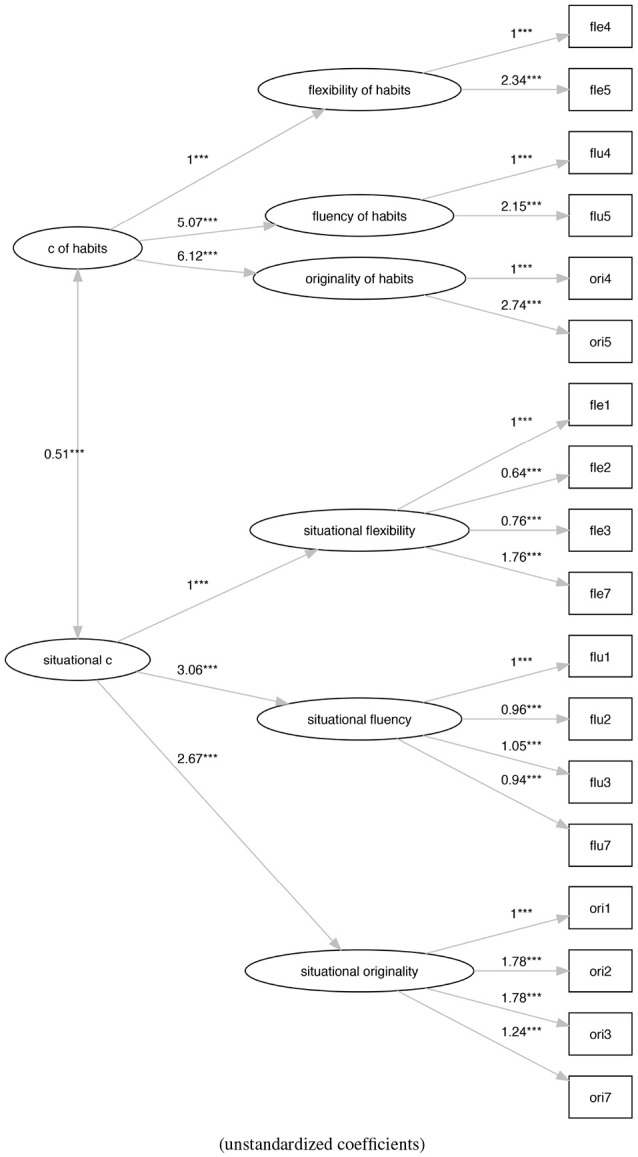
The social practice-based re-arranging of the original Torrance factor model (M2). **p < 0.01, ***p < 0.001.

Model (M2) (Figure 5) is a re-arrangement of the model M0 where we split the latent variables of fluency, flexibility, and originality into two groups according to our description in the section 3.2.1. The former group starts from the values collected from the tasks (1), (2), (3), and (7) to define what we call the “situational creativity.” The latter use results of the tasks (5) and (6) to define what we call the “creativity of habits.”

**Figure 6:**
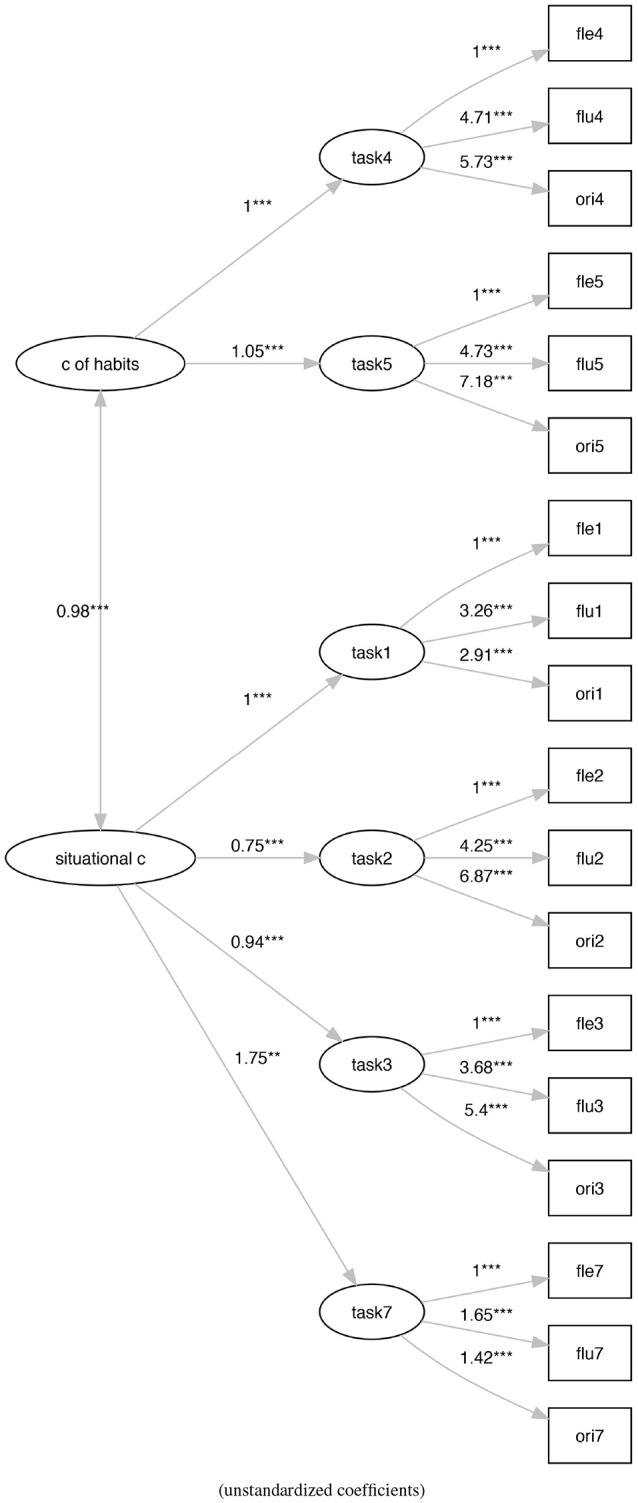
The social practice-based re-arranging of the tasks-based Torrance factor model (M3). **p < 0.01, ***p < 0.001.

Similarly, model (M3) (Figure 6) is a re-arrangement of the model M1 where we split the tasks' latent variables into two groups to define to the proposed two types of creativity.

These models refer to three different measurement models; in particular, the M0 model refers to the original measurement model with the three latent factors, flexibility (α = 0.66), fluidity (α = 0.78), and originality (α = 0.73), which show a good reliability.

The measurement model of M2 is based on the extraction of six latent factors whose Cronbach alphas that vary from a minimum value of 0.51 to a maximum value of 0.80. Finally, the M1 and M3 models share the measurement model based on six latent factors corresponding to the tasks of the Torrance; the Cronbach alphas vary from a minimum value of 0.78 to a maximum value of 0.84.

**Figure 7:**
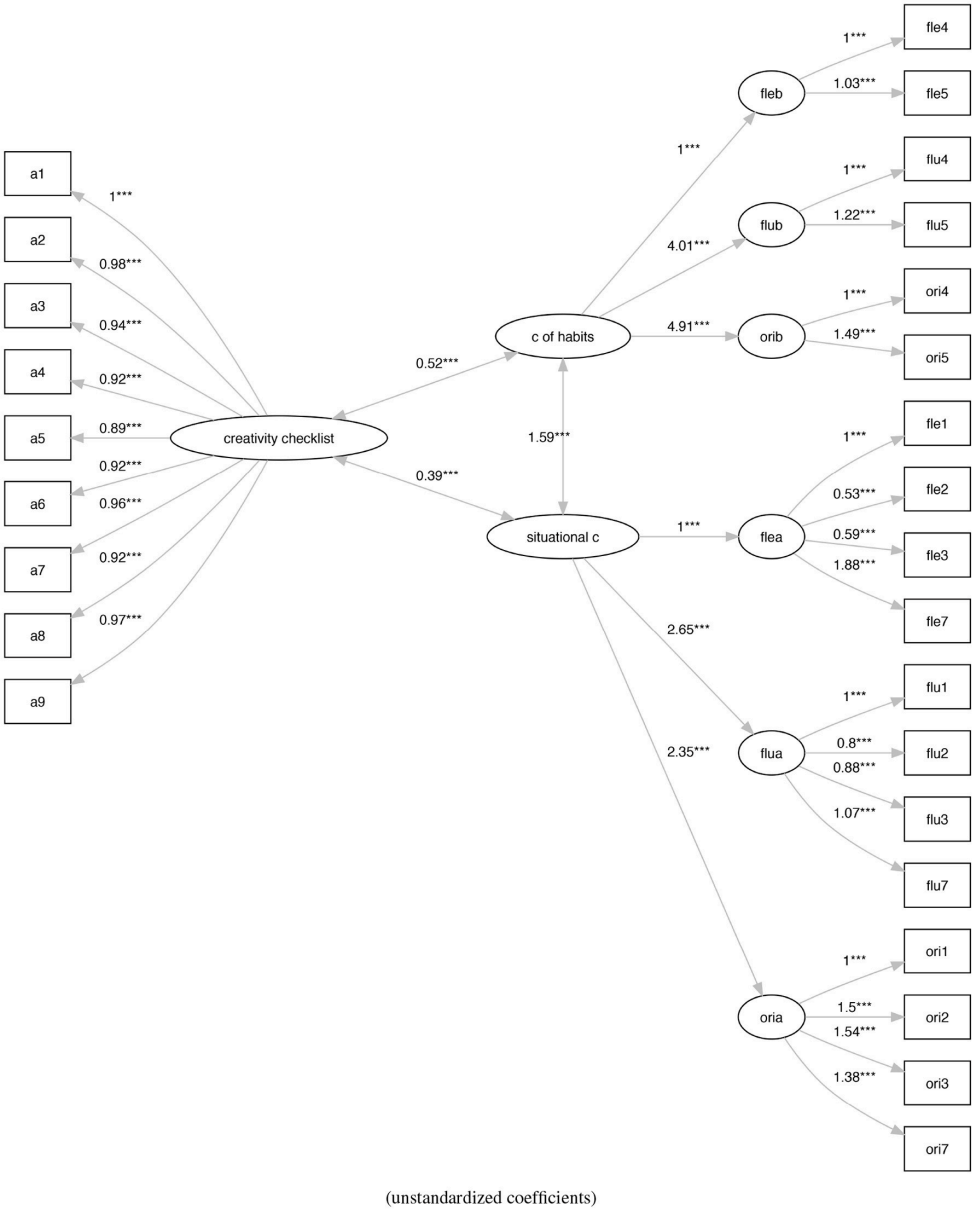
The analysis of social practice-based re-arranging of the original Torrance model with the Creativity Checklist (M4). ***p < 0.001.

**Figure 8:**
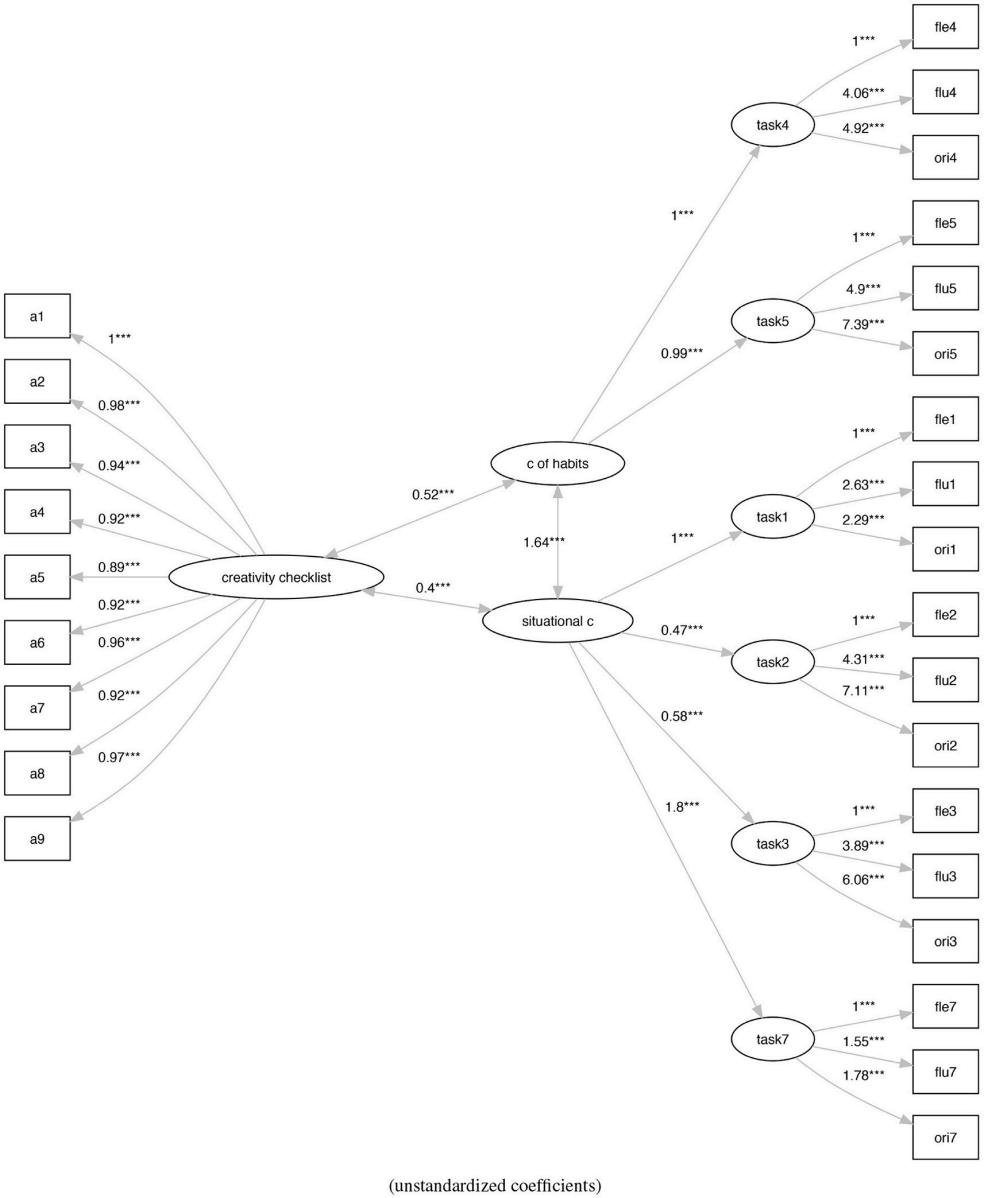
The analysis of social practice-based re-arranging of the tasks-based Torrance model with the Creativity Checklist (M5). ***p < 0.001.

Finally, to understand the nature of these two types of creativity, we linked the M2 and M3 models with an external measure of creativity, the Creativity Checklist, defining the models M4 (Figure 7) and M5 (Figure 8).

### 4.3. Model FIT Comparison

In Table 4 we report the original fit and the scaled fit, which originated from the analysis of the four models. The scaled fits were obtained using a maximum likelihood estimation with robust standard errors and a Satorra-Bentler scaled test statistic Satorra and Bentler (1994, 2011), which adjusts downward the value of the standard model chi-square from standard ML estimation by an amount that reflects the degree of kurtosis (Kline, 2012).

**Table 4:** Fit indexes of the analyzed Torrance factors models.

		**Chisq**	**Df**	**Chisq/df**	**Cfi**	**Tli**	**Rmsea**
M0		2235.24	132.00	16.93	0.40	0.31	0.29
	scaled	1532.23	132.00	11.61	0.31	0.20	0.23
M1		460.49	129.00	3.57	0.91	0.89	0.11
	scaled	295.28	129.00	2.29	0.92	0.90	0.08
M2		1707.43	128.00	13.34	0.55	0.47	0.25
	scaled	1091.48	128.00	8.53	0.53	0.43	0.20
M3		438.73	128.00	3.43	0.91	0.89	0.11
	scaled	283.27	128.00	2.21	0.92	0.91	0.08

Model M0 (Figure 3) shows a bad fit (ratio of χ^2^ to *df* = 11.61, CFI = 0.31, TLI = 0.20, RMSEA = 0.23), not verifying any of the generally accepted cutoff criteria (Schreiber et al., 2006). On the contrary, model M1 (Figure 4) shows an acceptable fit (ratio of χ^2^ to *df* = 2.29, CFI = 0.92, TLI = 0.90, RMSEA = 0.08), even if does not all the cutoff criteria are satisfied. Also, model M2 (Figure 5) shows a bad fit (ratio of χ^2^ to *df* = 8.53, CFI = 0.53,TLI = 0.43, RMSEA = 0.20), not verifying any of the generally accepted cutoff criteria. Model M3 (Figure 6) shows an acceptable fit (ratio of χ^2^ to *df* = 2.21, CFI = 0.92, TLI = 0.91, RMSEA = 0.08), even if does not all the cutoff criteria are satisfied.

The models defined according the theory of social practices show an improvement with respect to the starting ones. Model M2, even though it does not show a good fit, is nevertheless better than model M0. Moreover, as shown in the table, model M3 showed improvement not only compared to model M1 (Δχ^2^(1) = 7.4235, *p* < 0.01), but also compared to all models.

Finally, in Table 5 we report the original fit and the scaled fit, which originated from the analysis of the two models in which we check the relationship among the two types of creativity and the external measure defined by the Creativity Checklist.

**Table 5:** Fit indexes of the proposed conceptual models.

		**Chisq**	**Df**	**Chi/df**	**Cfi**	**Tli**	**Rmsea**
M4		692.48	315.00	2.20	0.99	0.98	0.08
	scaled	736.85	315.00	2.34	0.93	0.92	0.08
M5		379.28	315.00	1.20	1.00	1.00	0.03
	scaled	504.60	315.00	1.60	0.97	0.96	0.06

While models shows an acceptable fit, model M5 (Figure 8) (ratio of χ^2^ to *df* = 1.6, CFI = 0.97, TLI = 0.96, RMSEA = 0.06) shows a better fit with respect to model M4 (Figure 7) (ratio of χ^2^ to *df* = 2.34, CFI = 0.93, TLI = 0.92, RMSEA = 0.08).

## 5. A Discussion About the Implications of the Study For the Development of Creative Social Artificial Agents

The results discussed in section 4 confirm that the grouping of the activities of the Torrance test according to the proposed analysis corresponds to two different (but related) latent factors, both of which are correlated to the creativity measured by the Creativity Checklist. This is the case whether we carry out this rearrangement starting from the original model of the Torrance, or if we do it on the activity-based measurement model.

Therefore, the results seem to confirm the hypothesis that creativity is intrinsic in practices of thinking and acting in society, and that in particular, focusing on this dependence, it is possible to depict the two aforementioned typologies of creativity.

Most importantly, the analysis conducted so far offers an interesting perspective regarding the creativity mechanisms, that can be a valid starting point to introduce creative behavior in a computational agent. As noted in the Introduction, we claim that adding a sort of creative process can improve the agent's social behavior. This is our long-term goal, a goal resulting from the need to have artificial agents able to manage the variability of social contexts and adapt their behavior dynamically to the different social situations.

In this section, we deepen the explanation of what we intend with these typologies of creativity, that we outlined in section 2, and, supported by the results and by some examples extracted from the study carried out in the schools, we will discuss possible ways to introduce these two ways of being creative in a social agent, taking inspiration from existing approaches to computational creativity (Boden, 2004; Wiggins, 2006a,b; Augello et al., 2014, 2016b; Olteţeanu and Falomir, 2016; Kelly and Gero, 2017).

To have a clearer vision of how a social, creative behavior can be implemented in an artificial agent, we rely on a conceptualization of social practices introduced in (Dignum and Dignum, 2014; Dignum et al., 2015; Augello et al., 2016a; Dignum, 2018). According to this model, a practice can be characterized by a structure with the following elements: Context, Activities, Meanings, and Expectations.

***Context***

- *Actors* are all people and autonomous systems involved, that have capability to reason and (inter)act. This indicates the other agents that are expected to fulfill a part in the practice.
- *Roles* describe the competencies and expectations about a certain type of actors. Thus a lecturer is expected to deliver the presentation.
- *Resources* are objects that are used by the actions in the practice such as seats, projector, screen, etc. So, they are assumed to be available both for standard actions and for the planning within the practice.
- *Affordances* are the properties of the context that permit social actions and depend on the match between context conditions and actor characteristics.
- *Places* indicates where all objects and actors are usually located relatively to each other, in space or time: Seats in a lecture theater all face the front of the room, etc.

***Meaning***

- *Purpose* determines the social interpretation of actions and of certain physical situations.
- *Promotes* indicates the values that are promoted (or demoted, by promoting the opposite) by the social practice.
- *Counts-as* are rules of the type “X counts as Y in C” linking brute facts (X) and institutional facts (Y) in the context (C). For example, in a voting place, filling out a ballot counts as a vote.

***Expectations***

- *Plan patterns* describe usual patterns of actions defined by the landmarks that are expected to occur.
- *Norms* describe the rules of (expected) behavior within the practice, as statements of the form ADIC or ADICO (Crawford and Ostrom, 1995)^3^.
- *Strategies* indicate the possible activities that are expected within the practice. Not all activities need to be performed! They are meant as potential courses of action. Strategies are specified as AIC statements (Crawford and Ostrom, 1995)
- *Start condition*, or trigger, indicating how social practice starts
- *Duration*, or End condition, indicating how social practice ends

***Activities***

- *Possible actions* describes the expected actions by actors in the social practice
- *Requirements* indicate the type of capabilities or competences that the agent is expected to have in order to perform the activities within this practice.

It should be pointed out that the model of social practices (Dignum and Dignum, 2014; Dignum et al., 2015; Augello et al., 2016a; Dignum, 2018) is a conceptual model that does not correspond to a unique implementation, but which represents a framework from which it is possible to define different agent architectures.

### 5.1. Toward Computational Situational Creativity

The first type of creative process emerging from the our study is the situational one that, as explained in section 4.2, has been observed in Tasks (1), (2), (3), and (7).

**Figure 9:**
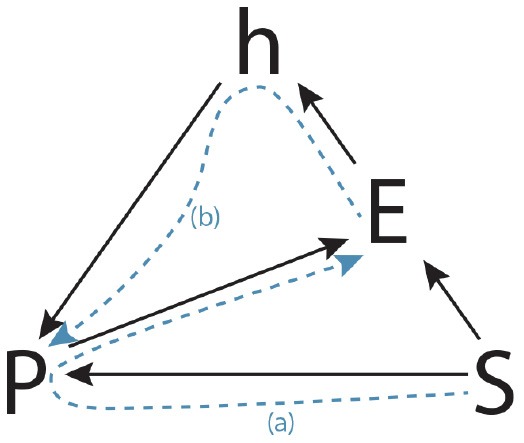
Situational creativity path in the SHaPE model.

Referring to the SHaPE model, we can represent this creative process as a path in which it is possible to identify two distinct phases. In a first phase, the situation leads to the activation of a specific practice (“path a” in Figure 9). Even if the situation triggers a practice, it would be necessary for the agent to introduce some variations (“path b” in Figure 9), by exploiting his habits.

For example, the practice raised by Tasks (1), (2), and (3), related to a story schema, is depicted in Table 6 according to the model introduced above. As shown in the table, some elements are fixed, since they are clearly interpreted in the drawing, while other elements, highlighted in bold, can be managed by the students more or less creatively to “generate” different versions of the story/practice.

**Table 6:** The social practice description of the tasks (1), (2), and (3) of TTCT.

**Context**	
Actors	The character in the picture, body features of the character (ears,face expression, eyes, etc.)
Roles	Role of the character
Resources	Water, ground, mirrored image of the character, clothes (hat, shoes, etc.)
Places	The character is near the water, place
**Meanings**	
Purpose	Of actions and physical situations
Promote	The moral of the story
**Expectations**	
Plan pattern	Patterns of ordered set of actions to reach a goal
Norms	Rules that describe expected behaviours in the practice
Start Condition	Indicating how social practice starts
End Condition or Duration	Indicating how social practice ends, expected durations of actions and plans
**Activities**	
Possible Actions	Actions of the character, actions of the actors that are not in the picture
Requirements	The type of capabilities or competences that the agent is expected to have in order to perform the activities within this practice

In a similar situation, an artificial agent can exploit the completion/variation of the components of the practice that could miss, or must be replaced or adapted according to the specific situation. For example, previously we said that a practice indicates the resources necessary for its implementation, including their affordances. If some resources are missing, the agent can play that practice by replacing them with other objects that have overlapping features. In this case, the agent manages a problem of object replacement or object composition, an argument of great interest in computational creativity (Olteţeanu and Falomir, 2016); the research about structural, functional and behavioral analogy of object can be applied to tackle this task. The agent can do the same with the actors and the roles they play - for example finding other actors that are capable of playing the same role. Even the plan patterns section could be creatively re-arranged. Plan patterns describe usual patterns of actions (Bresciani et al., 2004) defined by the landmarks that are expected to occur. The use of landmarks to delimit possible courses of actions gives a lot of flexibility to the agent.

Situational creativity could also be achieved using the standard Wiggins approach (Wiggins, 2006a,b) of searching in a space of solutions. According to Wiggins, it is possible to think of social practices as the nodes of the research space, where the universe *U* will be composed of all possible social practices known by the agent. Because social practices are connected in a kind of hierarchy through generalizations and abstractions one can imagine traversing this network using operators that first generalize the social practice at hand and then instantiating it in different ways again. Thus, we get variations on the social practice that differ in more aspects if we go higher up the social practice tree first before coming down in another branch of the tree again. This approach requires that we have an ordering of elements that are more or less central to the meaning of the social practice. Changing any of the central elements will create a radically different practice, while changing any of the other elements can be seen as creating different variations of the same practice. For Example, making a piece of art through painting can be changed in drawing, collage, etc., which are all two-dimensional and create products that can be hung on a wall. When we change from painting to claying we go from 2-dimensional art to 3-dimensional art, which is different in a more radical way. If we go from producing physical products to e.g., music we make an even more radical change.

### 5.2. Toward Computational Creativity of Habit

The creativity of habits is, in our vision, a creative process that is triggered when an agent does not recall a practice strictly bound to the specific situation, but rather he defines a new one. This missed correspondence can be due to the fact that the situation is not well defined or it is unexpected, but in some cases, it could be a decision of an agent to experiment something different from an ordinary behavior. The Torrance test stimulates such a behavior by relaxing the constraints of a situation in Tasks (4) and (5), by asking the students to conceive novel uses of boxes or to design a more entertaining toy. The absence of constraints or strong expectations in the situation allowed the students to, in some sense, diverge from ordinary practices of actions.

**Figure 10:**
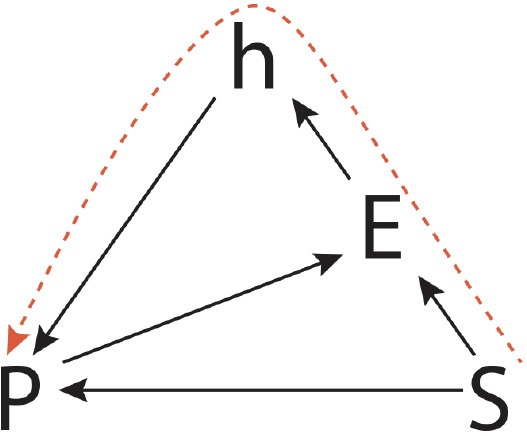
Creativity of habits path in the SHaPE model.

Referring to the SHaPE model, we can represent this process as the path depicted in Figure 10, where from a given situation the agent does not recall a practice but instead, gains from his habits to define a new practice, where the habits have been consolidated throughout his practical experiences. A student by imaging a box, or by examining and manipulating a real object represented by the elephant toy in the test, thanks to his habits, can rely on their peculiar features and affordances, to generate new practices involving such objects. It is interesting that in the Torrance tasks related to such a process, the students have been evaluated more creative. It is not a simple adaptation of a practice to the situation, but the generative power of habits is stimulated to find new solutions. It is possible to highlight also that, in these cases, the students show a predisposition to perform conveyances and/or combinations between different practices, because of their habits. This is the type of creativity that will lead a person to recognize in a cloud those features that resemble a cat or to recognize in a box a regularity of features that lead him to compare it to the structure of a theater, or the interior of a car.

Such a creativity is more intriguing and challenging to simulate in an artificial agent. It is certainly possible to make an analogy between the consolidation of habits of a human being and the process of learning from data for an artificial agent trained with a machine learning algorithm. Deep learning mechanisms capable of highlighting regularity in the agent's experiences could be investigated. The agent, during his experiences, could learn particular structures in what it observes. What he learned, even if not yet structured in a practice, constitutes a potential, a predisposition, that can be considered to manage new or less constrained situations. The challenge is first of all formalizing and using computationally what Bourdieu calls the habit, and then define generative algorithms that allow an agent equipped with the social practice model, to generate new practices gaining from his habits not explicitly connected to the analyzed situation.

## 6. Conclusion

In this work, we claim that to improve the agent's social behavior it is necessary to add a sort of creative process that allows artificial agents to cope with the variability of social context. According to the social practice theory, an agent should be able to deliberate within a practice, but also to dynamically transform or generate new practices starting from his experience. The SHaPE model introduced in this work, describing the relationship between the concept of social practice and the two related concepts of situation and habits, allowed us to hypothesize two possible creative processes.

To confirm our assumption, we analyzed the Torrance Test of Creative Thinking in the light of the SHaPE model. The results show that the tasks of the TTCT could be organized into two groups that correspond to the two different ways of looking at the creativity.

This study represents a first step in the analysis of creative processes within social practice theory. Further studies are needed to confirm the results obtained and to verify if these results are replicable with other tools for measuring creativity.

For example, we plan to test our hypothesis on the *situational creativity* and the *creativity of habit* in the context of the non-verbal form of TTCT. In particular, referring to the way the tasks of the figural forms of the test are structured, we hypothesize that these tasks will allow us to explore more deeply and in even greater detail the processes underlying creativity of habit.

To the aim of this work, we did not “enter” into the evaluation criteria of the Torrance, in fact, we strictly follow the indications of the evaluation manual. Nevertheless, a possible revision of TTCT according to the operationalization of the social practice model could be proposed, for example, changing the organization of the evaluation categories for each task. Of course, this would lead to the definition of a new tool for evaluating creativity that would require a new validation.

In addition, the creation of *ad-hoc* experimental designs - based on tasks that can better separate the two types of creativity - may be necessary to improve our understanding of the two processes. Finally, referring to the computational side, we discussed possible ways to introduce the two forms of being creative in a social agent, relying on a conceptualization of social practices. The computational approaches of creativity outlined in this work will be used to improve the aforementioned social agent's framework (Dignum and Dignum, 2014; Dignum et al., 2015; Augello et al., 2016a). The creative social agent will be used to check if and how the resulting framework can be used to get more insights into the elements of the creative process and its relationship to social practices in human contexts.

## Ethics Statement

This study was carried out in accordance with the recommendations of COMITATO BIOETICO A.O.U.P. P. Giaccone. The protocol was approved by the COMITATO BIOETICO A.O.U.P. P. Giaccone. All subjects gave written informed consent in accordance with the Declaration of Helsinki.

## Author Contributions

GC contributed to defining the theoretical background of the work, analyzing creativity in light of social practice theory. He also collaborated to collecting and scoring the tests. MG contributed to defining the original idea of the paper and the related research design. He collaborated to the praxeology interpretation of the TTCT and the statistical analysis of the results. AA contributed to defining the theoretical background of the work for what concerns the computational creativity aspects. She collaborated to the praxeology interpretation of the TTCT and collaborated to collecting and scoring the tests. SO contributed to the research design by analyzing and selecting the instruments used in the research. She participated in the collection and scoring of the tests. MA supported the definition of the research design and the writing of the document. FD participated in the definition of the theoretical background of the work and the analysis of the TTCT according to the Social Practice Model.

### Conflict of Interest Statement

The authors declare that the research was conducted in the absence of any commercial or financial relationships that could be construed as a potential conflict of interest.

## Footnotes

^1^The expression “situational creativity” has already been used in literature in different contexts. Goswami (Goswami, 1996) defines situational creativity as that type of creativity that involves the production of new meanings from an already known context. Schermerhorn (Schermerhorn Jr, 2009), within the field of research on management, describes some elements (e.g., management support) in terms of situational creativity drivers, components able to unlock the creative potential of members of a team. However, despite previous uses, our use of situational creativity places, in an original way, this expression in the dimension of the theory of social practices. Here the concept of situation and the term “situational” has some characteristics that go beyond the simple identification of it with the context , gives a detailed connotation to a specific path of creativity and is closely linked to key the concepts of habit, practice and experience (cf. section 2).

^2^The evaluator locates students' sentences in a list of categories of flexibility reported in the evaluation manual. For instance, for tasks (1), (2) and (3) the categories are the following: Clothing/Costume, Context/Environment, Physical action outside the picture, Physical action in picture, Ornamental objects, Emotions, Family, Ethnic Matters, Location, Magic, Occupation, Characters in picture, Characters outside the picture, Time, Weather/Natural disaster, Whole picture. For each category, moreover, the evaluation manual reports a series of sample propositions for facilitating the classification.

^3^ADICO is the complete form to describe norms. It stands for *Attributes* that indicate for whom the norm is applicable, *Deontic* for the deontic operator (Obligated,Forbidden,Permitted), the *aIm* indicating what action or state the norms is directed at, *Conditions* indicating when the norm is applicable and *Or else* indicating the sanctions when the norm is violated. ADICO has all components, ADIC is for social norms that do not have explicit sanctions and AIC for general conventions that are not normative.
